# Examination of concomitant glenohumeral pathologies in patients treated arthroscopically for calcific tendinitis of the shoulder and implications for routine diagnostic joint exploration

**DOI:** 10.1186/s12891-017-1839-z

**Published:** 2017-11-21

**Authors:** Gernot Lang, Kaywan Izadpanah, Eva Johanna Kubosch, Dirk Maier, Norbert Südkamp, Peter Ogon

**Affiliations:** 1 Department of Orthopedics and Trauma Surgery, Medical Center - Albert-Ludwigs-University of Freiburg, Faculty of Medicine, Albert-Ludwigs-University of Freiburg, Hugstetter Strasse 55, 79106 Freiburg, Germany; 2 Center of Orthopedic Sports Medicine Freiburg, Breisacher Strasse 84, 79110 Freiburg, Germany

**Keywords:** Calcific tendinitis, Rotator cuff, Shoulder arthroscopy, Postoperative recovery, Outcome factors, Shoulder, Degeneration, Glenohumeral, Osteoarthritis, Diagnostic, Tendinopathy

## Abstract

**Background:**

Glenohumeral exploration is routinely performed during arthroscopic removal of rotator cuff calcifications in patients with calcific tendinitis of the shoulder (CTS). However, evidence on the prevalence of intraarticular co-pathologies is lacking and the benefit of glenohumeral exploration remains elusive. The aim of the present study was to assess and quantify intraoperative pathologies during arthroscopic removal of rotator cuff calcifications in order to determine whether standardized diagnostic glenohumeral exploration appears justified in CTS patients.

**Methods:**

One hundred forty five patients undergoing arthroscopic removal of calcific depots (CD) that failed conservative treatment were included in a retrospective cohort study. Radiographic parameters including number/localization of calcifications and acromial types, intraoperative arthroscopic findings such as configuration of glenohumeral ligaments, articular cartilage injuries, and characteristics of calcifications and sonographic parameters (characteristics/localization of calcification) were recorded.

**Results:**

One hundred forty five patients were analyzed. All CDs were removed by elimination with a blunt hook probe via “squeeze-and-stir-technique” assessed postoperatively via conventional X-rays. Neither subacromial decompression nor refixation of the rotator cuff were performed in any patient. Prevalence of glenohumeral co-pathologies, such as partial tears of the proximal biceps tendon (2.1%), superior labral tears from anterior to posterior (SLAP) lesions (1.4%), and/or partial rotator cuff tears (0.7%) was low. Most frequently, glenohumeral articular cartilage was either entirely intact (ICRS grade 0 (humeral head/glenoid): 46%/48%) or showed very mild degenerative changes (ICRS grade 1: 30%/26%). Two patients (1.3%) required intraarticular surgical treatment due to a SLAP lesion type III (*n* = 1) and an intraarticular rupture of CD (n = 1).

**Conclusions:**

Routine diagnostic glenohumeral exploration does not appear beneficial in arthroscopic treatment of CTS due to the low prevalence of intraarticular pathologies which most frequently do not require surgical treatment. Exploration of the glenohumeral joint in arthroscopic removal of CD should only be performed in case of founded suspicion of relevant concomitant intraarticular pathologies.

## Background

Calcific tendinitis of the shoulder (CTS) is a common musculoskeletal disorder (2.7% to 20% of the population), usually affecting women more often than men [1, 2]. The pathogenesis of CTS is still poorly understood. To date, several theories on the development of CTS exist: while some authors propose that calcification of a tendon might occur due to vascular ischemia, repetitive micro trauma, or cellular necrosis of tissue, others believe that CTS might be an active cell mediated process (based on multiple factors, i.e. genetic disposition, environmental factors, metabolic disorders, etc.) resulting in alterations of cell and extracellular matrix differentiation [3]. Most frequently, CTS can be treated conservatively with satisfying outcome. Ultrasound-guided (US) needling and extracorporeal shock wave therapy (ESWT) have recently emerged as alternative therapies in CTS patients and demonstrated good to excellent clinical outcome [1, 4, 5]. When primary and secondary conservative treatments fail due to chronicity of symptoms, eventually more invasive treatment modalities are indicated in 10% to 15% of patients. To date, shoulder arthroscopy has been the preferred therapy as surgical treatment option, as it provides minimal invasive access to the glenohumeral joint, rotator cuff, and subacromial space, allowing for efficient removal of calcific deposits (CD) under direct visualization. In these cases, surgical procedures have proven to relief pain and improve function significantly [6–8]. Although arthroscopy includes a significant reduction of approach-related comorbidity compared to open procedures, aggressive arthroscopic CD removal often results in relevant rotator cuff defects including a variety of complications, such as arthrofibrosis and/or shoulder stiffness, conditions that potentially require additional surgical treatment and/or prolonged rehabilitation [7, 9–12]. Furthermore, shoulder arthroscopy is associated with certain inherent complications which can lead to devastating outcome [13–17]. In addition to that, the extent of CD removal as well as the clinical benefit of subacromial decompression and/or glenohumeral joint exploration remain elusive. Moreover, glenohumeral exploration accounts for additional operative time, which potentially translates into increased perioperative risk for complications as well as additional costs. As there is no evidence supporting that patients with CTS benefit from glenohumeral exploration during arthroscopic removal of calcifications, it is difficult to justify the supplemental approach to the joint.

Arthroscopic treatment of CTS usually starts with diagnostic glenohumeral joint exploration, followed by CD removal through a direct subacromial approach. Recently, Sirveaux et al. proposed that diagnostic glenohumeral arthroscopy should not be performed in routine manner when treating patients with CTS [18]. In this retrospective, comparative study, the cohort of 32 patients undergoing additional diagnostic glenohumeral arthroscopy showed significantly prolonged postoperative pain (11 weeks vs. 6 weeks) and a significant latency in return-to-work (12 weeks vs. 5 weeks). The authors concluded, that the additional intervention of diagnostic glenohumeral joint exploration would cause further harm rather than yielding improved clinical outcome [18]. The authors found concomitant pathologies in 5/32 (15.6%) cases. However, none of them required surgical intervention. In conclusion, the authors recommended excising as much of the CD as possible via a direct bursal approach without performing additional diagnostic glenohumeral arthroscopy. However, aggressive CD removal at expense of the affected rotator cuff’s integrity might negatively influence postoperative recovery and outcome. In addition to the retrospective study design with a limited case number, this issue might have substantially biased the result of the study of Sirveaux et al.. Previously, it was demonstrated that blunt arthroscopic CD removal preserving the integrity of the rotator cuff resulted in good to excellent outcome in 90% of patients [6, 19]. The authors tolerated minor remnant calcifications in favor of rotator cuff integrity. None of the patients received an additional rotator cuff repair. The remaining calcifications did not impair functional outcome and had spontaneously resolved until follow-up.

To date, there is minimal knowledge on the prevalence of concomitant pathologies in CTS. The study of Sirveaux et al. described a prevalence of 15.6% but the case number is too low for a conclusive assessment. Given the controversial findings and argumentation throughout literature, it still remains questionable whether diagnostic glenohumeral arthroscopy is justified in arthroscopic treatment of CTS.

Therefore, the purpose of this study was to investigate the prevalence and clinical significance of concomitant glenohumeral pathologies assessed during arthroscopic treatment of rotator cuff calcifications in order to determine whether diagnostic glenohumeral exploration appears justified in routine manner. We hypothesized a low prevalence of concomitant pathologies in patients with CTS and assumed that diagnostic glenohumeral exploration might not be required in a standardized fashion.

## Methods

### Study population

We conducted a descriptive retrospective cohort study of patients presenting with symptomatic chronic CTS undergoing arthroscopic removal of CD between 2008 and 2011 that failed at least six months of conservative treatment. Patients had been referred by a general practitioner, rheumatologists, or orthopedic surgeon for treatment. Most common clinical findings were persistent shoulder pain, functional disability and the presence of symptomatic rotator cuff calcifications. Further inclusion criteria were the presence of radiographically and sonographically determined calcifications within the rotator cuff and the presence of clinically symptomatic CTS of the shoulder. Patients with previous ipsilateral shoulder surgeries or shoulder comorbidities were excluded. Calcifications were confirmed preoperatively by conventional radiographs (anterior-posterior) and bilateral ultrasound examination.

### Preoperative localization of CD via quadrant technique

Preoperative ultrasound examination (1 to 2 days before surgery) of the rotator cuff was performed in a standardized manner by a board certified orthopedic surgeon who was experienced with musculoskeletal sonography. Calcific depositions were evaluated according to the quadrant technique within preoperative outpatient consultation [20]. The number of calcifications and dorsal attenuation were recorded accordingly. Additionally, ultrasound was used to assess the integrity of the rotator cuff and biceps tendon.

### Radiographic evaluation

Radiographic parameters (based on standard anterior-posterior (a.-p.) and outlet radiographic views) were measured at two time points: preoperatively (1 to 2 days before surgery) and postoperatively (within 2 to 3 days after surgery). The number of calcific lesions and localization of calcifications (medial/lateral) were assessed using standardized a.-p. films. Acromion morphology was classified according to Bigliani et al. on outlet radiographs [21]. Postoperative radiographs were performed to determine the amount of CD removal.

### Surgical procedure

All surgeries were performed by the same board-certified orthopedic surgeon experienced in shoulder arthroscopies (PO). Our surgical procedure of shoulder arthroscopy in patients with CTS has previously been described in detail [6]. Briefly, under general anesthesia patients were positioned laterally in a decubitus position with slight extension to the arm. Preoperatively, the exact localization of CD was determined by utilization of ultrasonographical mapping according to the quadrant technique [20]. As the patient’s arm was resting in the neutral position, the intraoperative anatomy was configured in the same exact fashion as performed for preoperative localization of the rotator cuff calcifications, which facilitated in situ localization of CD significantly. The calcifications were identified and marked with respect to the affected quadrants.

**Fig. 1:**
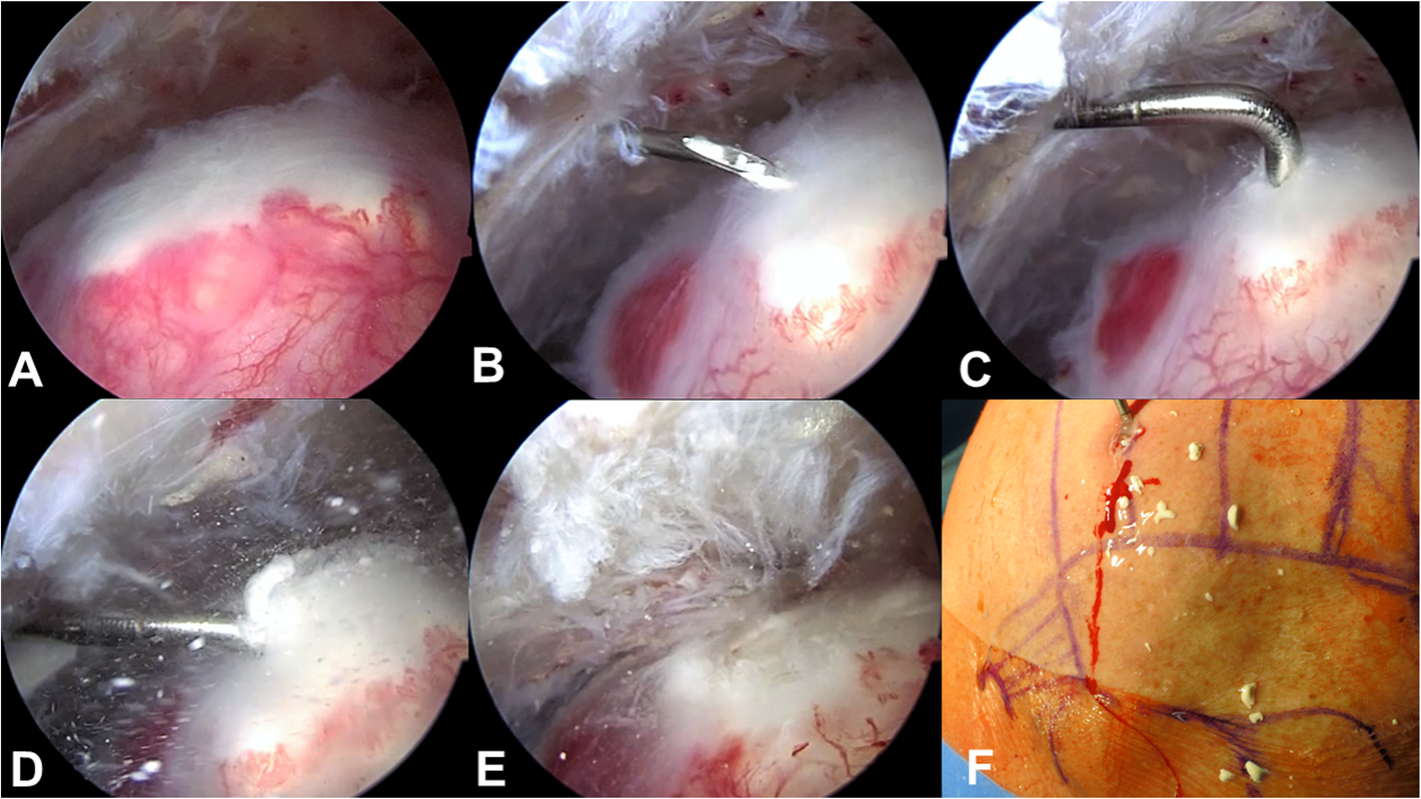
Surgical steps for minimally invasive arthroscopic removal of rotator cuff calcifications. **a**. Identification of the calcific deposit. **b.** Localization of the calcific deposit’s center via needling. **c**. and **d**. A blunt hook probe is carefully advanced in order to gently release and eliminate the calcifications. **d**. “Snowstorm-phenomenon” - initial release of calcific particles. **e**. “Calcific cave” after removal of calcifications **f**. Macroscopic demonstration of removed calcific deposits on the patient’s skin

A stab skin incision was made followed by careful advancement of the scope into the glenohumeral joint through a posterior portal. The diagnostic arthroscopy was carried out in a conventional manner to rule out relevant intraarticular concomitant pathologies. Then, the subacromial space was identified and exposed using the same approach. Additionally, a lateral approach was used to insert a blunt trocar and subsequently gain access to the bursa. As the bursa was frequently hypertrophic and/or hypervascularized due to the chronic proinflammatory stimulus of the irritating intraarticular calcifications (Fig. 1), we performed a partial bursectomy by utilization of the arthroscopic shaver. The respective CD was then identified via needling and the center of the calcification was localized and incised with a needle (Fig. 1b). Calcific remnants in the needle’s tip indicated correct localization. Hereafter, a blunt hook probe was carefully advanced in order to gently release and eliminate (squeeze-and-stir-technique) the calcifications (Fig. 1c, d). Additionally, the superficial membrane was elevated in order to flush the joint followed by intermittent squeezing of the CD with the probe. This procedure was repeated until the calcific deposit was removed in a blunt fashion (Fig. 1e). Finally, a thorough irrigation of the “calcific cave” was performed by utilization of a syringe to ensure proper elimination of small calcific particles. Finally, the continuity of the affected rotator cuff tendon was confirmed precisely (risk for underestimation of rotator cuff tears following CD removal) before the wound was closed. No tendon repair was performed after CD removal.

### Assessment of concomitant glenohumeral pathologies

**Fig. 2:**
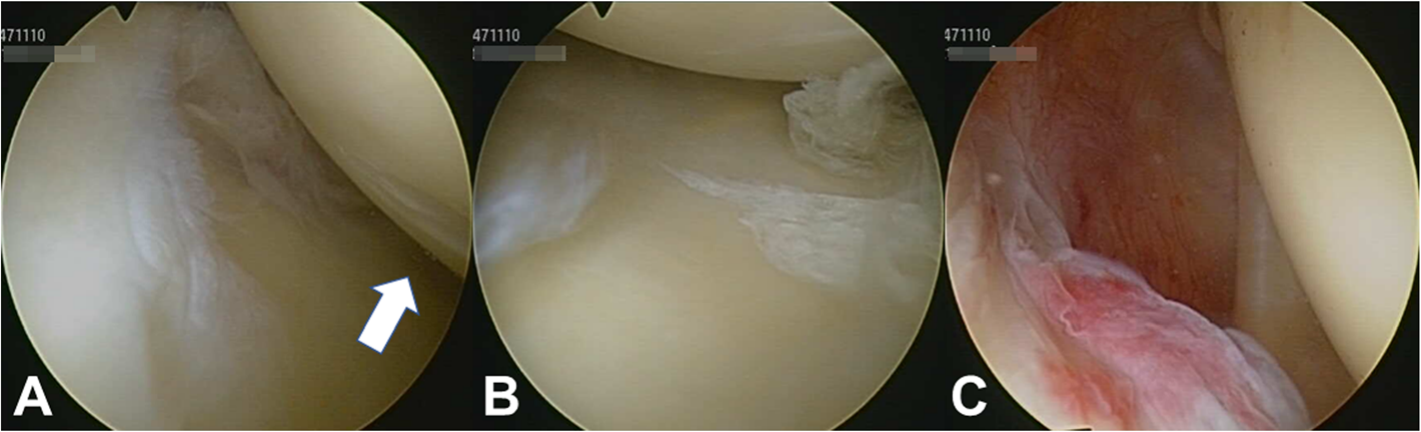
Cartilage lesions in a patient with calcifying tendinitis. **a**. Mild cartilage defect on the humeral head. **b**. Mild cartilage defect at the glenoid. **c**. Intact biceps and supraspinatus tendon

**Fig. 3:**
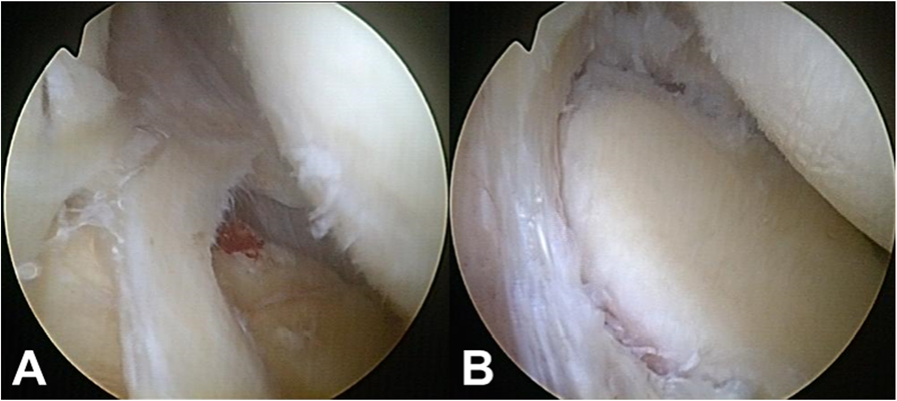
SLAP lesion type III according to Snyder et al. [18]. **a**. Intraoperative detection of a SLAP lesion type III which required surgical treatment. **b**. SLAP lesion type III after debridement and resection

Intraarticular concomitant pathologies were assessed within glenohumeral arthroscopy. The configuration of glenohumeral ligaments were evaluated according to Morgan et al. [22]. Glenohumeral cartilage injuries were assessed according to the International Cartilage Repair Society (ICRS, Table 1) cartilage injury classification (Fig. 2) [23]. Consistency and characteristics of calcifications were evaluated by macroscopic assessment and/or manipulation via blunt probe. Superior labral tears from anterior to posterior (SLAP lesions) were classified according to Snyder et al. (Fig. 3) [24]. Tears of the proximal biceps tendon were graded according to the thickness of injury (superficial, 25%, 50%, 75%, and full thickness rupture (100%). Rotator cuff lesions were classified according to Ellman et al. for partial tears and Bateman et al. for full thickness tears including indication of the affected tendon [25, 26].

**Table 1:** International Cartilage Repair Society (ICRS) Hyaline Cartilage Lesion Classification System [23]

Grade	Subgrade	Definition	Characteristics
0		Normal	Normal
1	A	Nearly Normal	Superficial lesions. Soft indentation
	B		A + and/or superficial fissures and cracks
2		Abnormal	Lesions extending down to <50% of cartilage depth
3	A	Severely Abnormal	Cartilage defects extending down to >50% of cartilage depth
	B		A + Cartilage defects extending down to calcified layer
	C		A + B + not extending through the subchondral bone
	D		A + B + C+ blisters are included
4		Severely Abnormal

### Statistical analysis

Continuous variables are shown as mean ± standard error of the mean. For categorical variables percentages were calculated. All analyses were performed using SPSS version 20 (IBM, Armonk, NY, USA).

### Ethical considerations

The study was approved by our local institutional review board and informed consent was obtained from all patients before surgery (protocol number: 598/16).

## Results

### Baseline characteristics

145 patients were enrolled in the study (66.2% female, Table 2). Mean age at surgery was 50.9 ± 8.9 years (range: 32–76). The major indication for arthroscopic removal of CD was symptomatic chronic CTS. The mean symptomatic time until patients decided to undergo surgical removal of CD was 3.5 years. 2.6% of patients had a previous shoulder surgery on the contralateral shoulder in the past. The most commonly treated side due to symptomatic CTS was the right shoulder in 54.5% of patients (Table 2). The dominant shoulder was affected in 33.1% (48/151).

**Table 2:** Patient demographics

Parameter		*N* = 145	%
Gender	Male	49	33.8
	Female	96	66.2
Mean age at surgery in years^1^	Max: 76 - Min: 32	50.9 ± 8.9	
ICRS cartilage injury – humeral head [23]	0	66	45.5
	1	43	29.7
	2	34	23.4
	3	0	0
	4	2	1.4
ICRS cartilage injury – glenoid [23]	0	70	48.3
	1	38	26.2
	2	35	24.1
	3	2	1.4
	4	0	0
Previous shoulder surgery	At the contralateral side	4	2.6
Intraarticular lesions		2	1.4
Intraarticular Co-pathology	Partial tear of the proximal biceps tendon (30% - 50% in relative width)	3	2.1
	SLAP lesion	2	1.4
	Interval rotator cuff lesion	1	0.7
Capsular type (n = 1 missing) [22]	1	135	93.1
	2	2	1.4
	3	7	4.8
	4	0	0
Intraoperative sublabral foramen	Yes	10	6.9
	No	131	90.3
	Buford complex	4	2.8
Side of injury	Left shoulder	66	45.5
	Right shoulder	79	54.5
	Dominant shoulder	48	33.1

Demographic characteristics of the study population

*ICRS* International Cartilage Repair Society, *SLAP* Superior labral tear from anterior to posterior, ^1^ Mean ± SD

### Preoperative radiographic assessment of calcifying tendinitis

Preoperative radiographic assessment of CTS was performed 1 to 2 days before surgery. As demonstrated in Tables 3, 72% of calcifications were localized laterally while 19.4% of calcific depots were found within the medial part of the rotator cuff. Few patients (8.3%) presented a bilateral configuration of the calcification (i.e. lateral +50% medial). Furthermore, the majority of patients (94.5%) featured a single calcific depot within the preoperative radiographic evaluation. Distribution of acromion morphology according to Bigliani et al., resulted in 31.7%, 59.3%, and 9% for a flat (type I), curved (type II) and hooked (type III) acromial shape (Table 3).

**Table 3:** Preoperative radiographic assessment of calcifying tendinitis

Parameter		N = 145	%
Localization (missing: n = 1)	Medial	28	19.4
	Lateral	104	72.2
	Lateral +50% medial	11	7.6
	Medial +50% lateral	1	0.7
Number of calcifications	1	137	94.5
	2	6	4.1
	3	0	0
	4	2	1.4
Acromion morphology [21]	1 – Flat	46	31.7
	2 – Curved	86	59.3
	3 – Hooked	13	9
Ultrasound – affected quadrants	1	115	79.3
	2	41	28.3
	3	10	6.9
	4	11	7.6
Ultrasound	Total number of lesions	139	
	Dorsal attenuation	118	81.4

### Concomitant glenohumeral pathologies

**Fig. 4:**
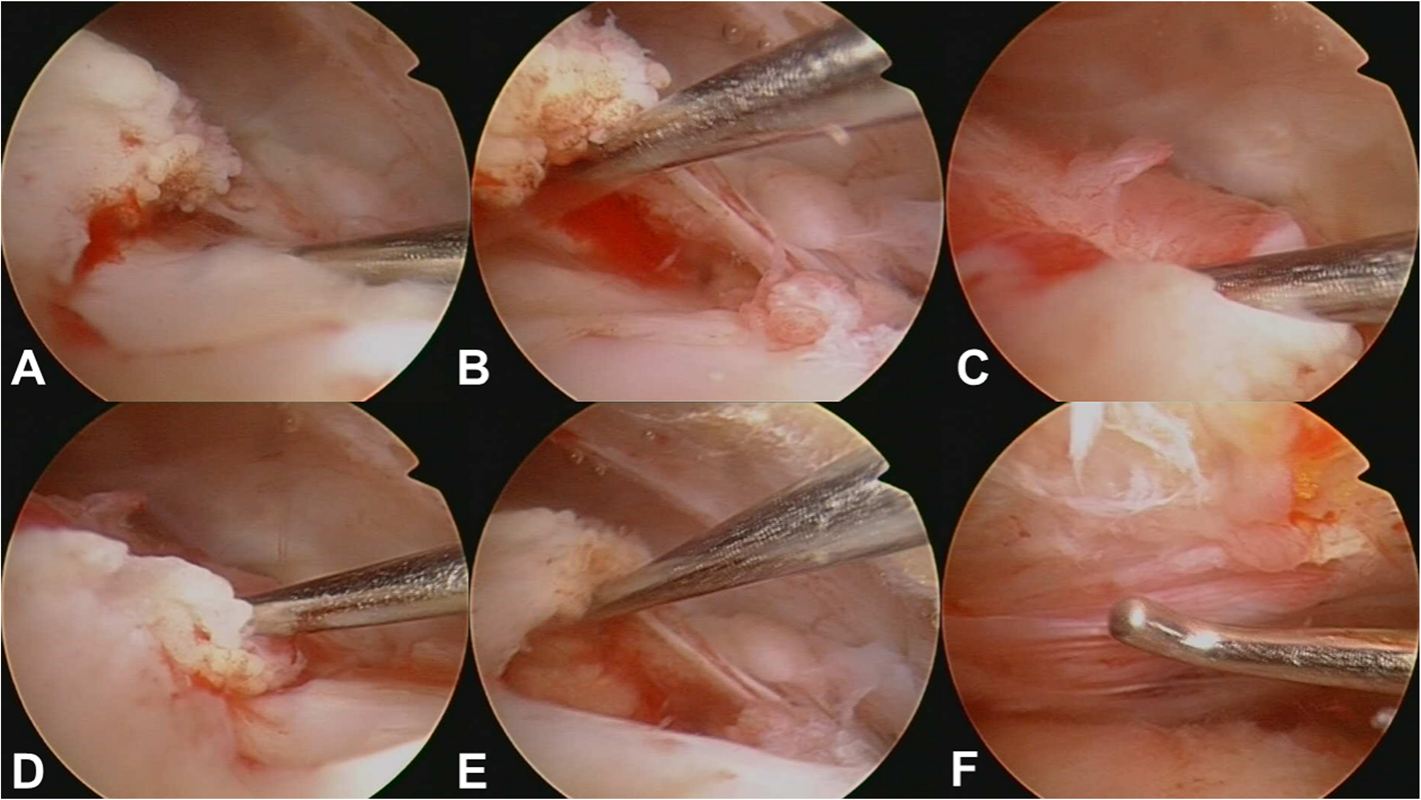
Acute bursal rupture of calcifications. **a**. – **f**. Intraoperative images display an acute intraarticular rupture of calcifications. The supraspinatus tendon demonstrates tears and fatty degeneration. A “cave” of the former calcifications remains as a partial bursal-sided tear

**Fig. 5:**
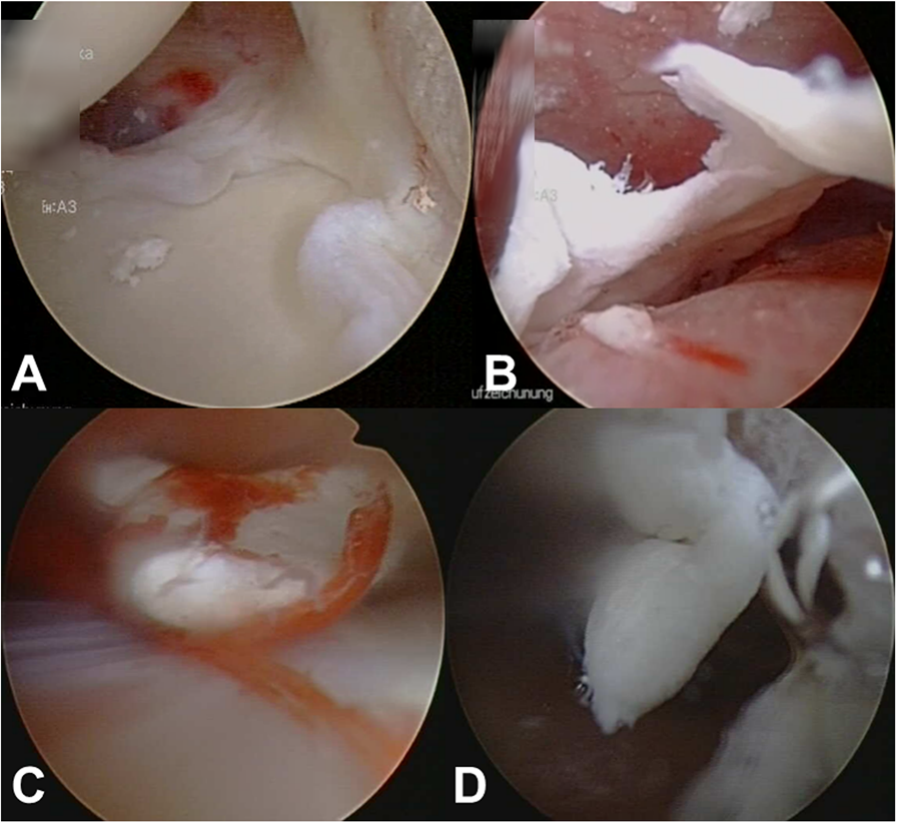
Intraoperative findings during removal of rotator cuff calcifications. **a**. Intraarticular calcific deposits in the glenohumeral joint space. **b**. - **d**. Large pieces of calcific deposits

The articular cartilage of the humeral head was intact in 45.5% of patients (Table 2). While mild cartilage injury at the humeral head (grade 1 and 2) was found in 29.7% and 23.4%, respectively, severe cartilage lesions were only detected in 1.4% (grade 4) of patients (Fig. 2). Moreover, 48.3% of glenoids did not reveal any cartilage injury at all. Mild cartilage lesions on glenoid articular surfaces were diagnosed in 26.2% (grade 1) and 24.1% (grade 2) of patients (Table 2). Severe glenoid lesions (grade 3) were recorded in 2 (1.4%) patients. Typical co-pathologies that were identified during arthroscopy were partial tears of the proximal biceps tendon (2.1%), SLAP lesions (1.4%), and rotator cuff defects (0.7%; Fig. 4). SLAP lesions and partial tears of the proximal biceps tendon were incidental findings. Furthermore, the glenohumeral capsular type was evaluated according to Morgan et al. (Table 2) [22]. The vast majority of our patients were classified as capsular type I (93.1%). Capsular type II and III were recorded in 1.4% and 4.8% of patients, respectively. Sublabral foramen were observed in 6.9% of cases, whereas over 90% of patients did not present any sublabral lesions. Two of our patients (1.4%) required specific glenohumeral surgical treatment due to a SLAP lesion type III and an intraarticular rupture of calcific deposits (Figs. 4, 5, and 3). As the other SLAP lesion in our study population was mild (type I) a debridement was not indicated. Neither did the patients with SLAP lesions complain about specific symptoms preoperatively nor have the SLAP lesions been detected via ultrasound (i.e. joint effusion) before surgery. One patient (0.7%) showed a tear of the rotator cuff interval without involvement of the subscapularis or supraspinatus tendon. CD removal was confirmed postoperatively by conventional X-rays.

### Intraoperative assessment of calcifying tendinitis

During arthroscopic removal of CD, rotator cuff calcifications typically presented with a tough and “tooth-paste”-like consistency (63.4%; mixed-forms were described); Table 4 and Figs. 1 and 5). We observed two cases (1.4%) of CD perforations into adjacent anatomical structures. One patient showed an extensive calcific bursitis as a result of CD rupture and perforation into the subacromial bursa. We observed CD perforation into the glenohumeral joint with both adhesive and free floating calcific components inside the joint in another patient.

**Table 4:** Intraoperative macroscopic characteristics of calcifying tendinitis

Parameter			N = 145	Percent
Consistency of calcification	Tooth paste		10	6.9
	Tough + Tooth paste		92	63.4
	Tough + Tooth paste + streaks		1	0.7
	Tough + Tooth paste + clods		1	0.7
	Snow		20	13.8
	Snow + flaky		12	8.3
	Snow + streaks		2	1.4
	Snow + toothpaste		3	2.1
	Red/brown “sauce”		1	0.7
	Calcification of bursa + snow		1	0.7
	Flakes on RC		1	0.7
	Cloudy		1	0.7
Structure of calcification	From very fine (1) to rough (5)	1	39	26.9
		2	1	0.7
		3	98	67.6
		4	2	1.4
		5	5	3.4

## Discussion

The purpose of the present study was to investigate whether glenohumeral arthroscopic exploration is justified in patients with CTS based on the prevalence of intraarticular pathologies that require surgical treatment. This study adds evidence that glenohumeral pathologies have a low prevalence in patients undergoing arthroscopic removal of rotator cuff calcifications. Outcome of this study suggests that a standardized diagnostic glenohumeral exploration may not be mandatory as a routine procedure during arthroscopic treatment of CTS. As far as we are aware, there is no previous investigation specifically analyzing the prevalence of intraarticular glenohumeral pathologies during arthroscopic removal of rotator cuff calcifications.

### Minimally invasive removal of rotator cuff calcifications

Most commonly, treatment of symptomatic CTS can be managed non-operatively (90% of patients) by physiotherapy, analgesics, and injections [27]. Surgical treatment is reserved for patients in which conservative therapy has failed, such as prolonged periods of functional disability, severe pain, and when calcific deposits do not resolve spontaneously. To date, most surgeons perform an arthroscopic removal of CD with/without subacromial decompression and glenohumeral exploration [7]. Recently, our group demonstrated that good to excellent results can be achieved in 90% of patients with blunt arthroscopic removal of calcific lesions, without performing a subacromial decompression. Therefore, we pursue a minimal-invasive strategy which rather tolerates minor remnant calcifications in favor of rotator cuff integrity [6]. In the present study, the vast majority of our patients did not present any intraarticular pathologies requiring surgical treatment. If intraarticular pathologies were detected, most likely these lesions did not cause any symptoms. Our results confirm the conclusion of Sirveaux et al., who compared clinical and radiographic outcome in patients with CTS treated either by CD removal alone via an arthroscopic bursal approach or CD removal combined with a standardized glenohumeral exploration [18]. The authors did not identify a single glenohumeral injury that required surgical treatment. Moreover, duration of postoperative pain and latency of return-to-work were significantly shorter in patients who received CD removal solely compared to patients that underwent CD removal including an additional glenohumeral exploration. After 6-month follow-up, functional outcome and radiographic CD disappearance did not reveal significant differences. Therefore, we agree on the authors’ statement, that a glenohumeral exploration can be avoided as the additional approach might include an increased risk for complications as well as it presumably leads rather to prolonged rehabilitation than clinical benefit. Furthermore, prolonged duration of surgery, increased postoperative pain and subsequently prolonged length of stay potentially cause higher costs [28, 29]. A glenohumeral exploration may be justified in specific scenarios: I.e. if a relatively large and consistent CD removal has been performed and an intra-articular assessment of the rotator cuff’s integrity is pursued. A thorough evaluation of the rotator cuff (especially of deep layers) may be facilitated by means of arthroscopic visualization. Nevertheless, for the majority of patients with CTS, an additional glenohumeral exploration seems to be an unnecessary and an expandable risk for complications as it contains an additional approach to the joint.

The amount of CD removal is another matter of debate: several authors favor a complete removal of CD while other authors presented good outcome with incomplete excision of CD [6, 7, 10, 30–32]. A total removal of CD may potentially result in a full thickness rotator cuff tear. Evidence is lacking whether an immediate reconstruction or no repair at all leads to superior outcome [6, 9, 31, 33, 34]. Last but not least, the size and consistency of CD lesions are highly important for the removal of rotator cuff calcifications. In the present study, the vast majority of CD lesions was either tough and tooth paste-like or presented as snowy powder, indicating that an easy removal is feasible without the application of intense forces.

In summary, instead of a standardized glenohumeral exploration within arthroscopic removal of CD lesions, we suggest a patient-specific treatment algorithm, that is individualized on the patient’s presenting complains in order to optimize the risk-benefit-ratio.

### Increased risk for complications due to glenohumeral exploration

CTS is predominantly an extraarticular phenomenon rather than a glenohumeral joint disease [1, 34]. Consequently, we expect an equivalent prevalence of glenohumeral pathologies in patients with CTS compared to healthy subjects. Certainly, an arthroscopic glenohumeral exploration provides the opportunity to detect intraarticular co-pathologies. However, our and previous data demonstrate that intraarticular co-pathologies in patients with CTS were hardly detected and moreover, if an intraarticular pathology was present, this almost never caused any procedural alteration [18]. Therefore, it is reasonable to question whether an additional glenohumeral arthroscopy can be justified in patients with CTS considering the increased risk for complications such as infection or shoulder stiffness against the lack of a true benefit through arthroscopy [18]?

The overall risk for complications in shoulder arthroscopy ranges between 4.8% and 10.6% [35–37]. Typical complications in patients having CTS are prolonged pain, secondary adhesive capsulitis, rotator cuff tears, ossifying tendinitis, and osteolysis of the greater tuberosity [38]. The incidence of a frozen shoulder after shoulder arthroscopy is 2% to 5% in the general population [39, 40]. Postoperative shoulder stiffness after rotator cuff repair ranges between 4.9% and 32.7% [41–43]. Shoulder stiffness is not well tolerated by patients with CTS; it does not resolve easily and may require long-term rehabilitation [44]. Reasons for shoulder stiffness are supposed to be the manipulation of capsule and/or residual calcium debris [6, 34]. Moreover, postoperative shoulder stiffness may be related to rotator cuff tear morphology, postoperative immobilization, glenohumeral adhesion, capsular contracture, or underlying predisposing patient comorbidities [42, 44, 45]. Reduction of approach-related comorbidity may potentially reduce complication rates and offers the opportunity to perform this procedure as an outpatient surgery that allows for immediate rehabilitation, which subsequently reduces the risk for postoperative stiffness [18]. Our current rate of postoperative arthrofibrosis is relatively low compared to previous studies – however, if the risk for postoperative arthrofibrosis can be further reduced without performing a glenohumeral approach while at the same time achieving equivalent outcome, one may assume that it might be reasonable to abstain from a glenohumeral exploration in routine fashion when treating patients with CTS arthroscopically [46]. However, this needs to be further confirmed in prospective comparative investigations.

Furthermore, infection following shoulder arthroscopy is another relevant risk factor. Pauzenberger et al. observed infections following arthroscopic rotator cuff repair in 0.009% [47]. The authors identified sex (male), age (≥60 years), and length of surgery (≥90 min) to be significantly associated with postoperative infection. Other groups reported overall infection rates after shoulder arthroscopy between 0.03% and 3.4% [37, 48–50]. Especially, joint infections due to Propionibacterium acne (P. acne) are currently controversially discussed in association with shoulder arthroscopy as conventional perioperative antibiotic- or preoperative prophylaxis do not seem to sufficiently decrease the risk for postoperative infections [47, 51]. Moreover, presurgical skin preparations do not entirely eliminate P. acne [52–54]. Current evidence suggests P. acne being the most frequent identified organism in shoulder infections [51, 55, 56]. Both, open and arthroscopic surgery provide approach related-opportunities for P. acne to be transferred from skin to deep layers and thus potentially causing glenohumeral joint infections [57]. Seth et al. observed differences in positive skin cultures contaminated by P. acne that were assessed before skin incision (15.8%) and directly before wound closure (40.4%), which underlines the association between length of surgery and potential risk for surgical side infections [58]. In order to minimize the risk for infections by P. acne, it is suggested to reduce the size and contamination of surgical approaches [59]. Due to the fact that glenohumeral joint infections are associated with disability and significant direct and indirect socioeconomic costs, we suggest to perform a glenohumeral joint exploration only if an intraarticular injury that requires surgical treatment is highly expected.

In general, the impact of glenohumeral exploration within arthroscopic removal of calcifications in CTS remains an under-investigated issue. To date, postoperative pain and latency of return-to-work were found to be significantly shorter in patients who received CD removal solely compared to patients that received an additional glenohumeral exploration [18]. It is reasonable that any additional manipulation/invasive maneuver during shoulder surgery might affect the clinical and/or functional outcome and potentially increases the likelihood of complications since this has already been demonstrated in various other surgical procedures [13, 37, 60, 61]. Investigations comparing complications of different arthroscopic approaches have not been performed yet. Sufficient data exist on 30-day readmission rates as well as risk factors for postoperative complications following shoulder arthroscopy as described by Shields et al. [45]. The authors found shoulder arthroscopy to have a 1% thirty-day complication rate. Age ≥ 60 years, operating room (OR) time ≥ 90 min, chronic obstructive pulmonary disease, inpatient status, disseminated cancer, and nicotine abuse are risk factors for postoperative complications [60]. These results have been confirmed by other authors such as Moody et al. who demonstrated, that prolonged operative time, more invasive and/or additional surgical approaches increase the risk for complications as well as health care expenditures [29, 60].

In the present study, intraarticular pathologies that needed surgical treatment have only been observed in few patients. For the majority of patients with CTS, an additional glenohumeral exploration might be an unnecessary risk and would most likely not translate into a clinical benefit. As patients undergoing surgery in CTS already reflect a “negative selection” due to failed conservative treatment, a reasonable risk-benefit-ratio by means of minimal approach-related comorbidity and perioperative risk for complications should be pursued. As there is currently no evidence supporting that patients with CTS benefit from glenohumeral explorations during arthroscopic removal of calcifications, the additional glenohumeral approach should only be performed in case of founded suspicion of relevant concomitant intraarticular pathologies.

### Limitations and strength

The retrospective study design is associated with certain limitations, such as loss of information (missing reports), heterogeneous preoperative conservative treatment, and unequal group power (i.e. gender, affected shoulder, etc.). Additionally, functional and clinical outcome data was not implemented into this study. Moreover, this study was of descriptive nature and did not include a control group. Nevertheless, as far as we are aware, there is no previous study specifically investigating intraarticular glenohumeral pathologies during CD removal in patients with calcifying tendinitis. Furthermore, a huge advantage of the present study is that all patients were treated by the same orthopedic surgeon resulting in high consistency and homogeneous evaluation of intraarticular conditions.

## Conclusion

Glenohumeral co-pathologies in CTS are rare and most commonly do not require surgical treatment. Routine diagnostic glenohumeral exploration does not appear mandatory in arthroscopic treatment of CTS. Exploration of the glenohumeral joint in arthroscopic removal of CD should only be performed when relevant intraarticular injuries with therapeutic relevance are highly expected.

## Abbreviations

CD: Calcific depots
CTS: Calcific tendinitis
OR: Operating room
SLAP: Superior labral tears from anterior to posterior lesions
